# Comparing Methods of Recruiting Spanish-Preferring Smokers in the United States: Findings from a Randomized Controlled Trial

**DOI:** 10.2196/19389

**Published:** 2020-08-14

**Authors:** Patricia Medina-Ramirez, Patricia Calixte-Civil, Lauren R Meltzer, Karen O Brandon, Ursula Martinez, Steven K Sutton, Cathy D Meade, Margaret M Byrne, Thomas H Brandon, Vani N Simmons

**Affiliations:** 1 Tobacco Research & Intervention Program Department of Health Outcomes & Behavior Moffitt Cancer Center & Research Institute Tampa, FL United States; 2 Department of Psychology University of South Florida Tampa, FL United States; 3 Biostatistics and Bioinformatics Department Moffitt Cancer Center & Research Institute Tampa, FL United States; 4 Department of Health Outcomes & Behavior Moffitt Cancer Center & Research Institute Tampa, FL United States; 5 College of Nursing University of South Florida Tampa, FL United States; 6 Department of Oncologic Sciences University of South Florida Tampa, FL United States

**Keywords:** Hispanic, Latino, smoking cessation intervention, randomized controlled trial, tobacco cigarette, recruitment, social media, Facebook, web banner ad

## Abstract

**Background:**

There is a pressing need to address the unacceptable disparities and underrepresentation of racial and ethnic minority groups, including Hispanics or Latinxs, in smoking cessation trials.

**Objective:**

Given the lack of research on recruitment strategies for this population, this study aims to assess effective recruitment methods based on enrollment and cost.

**Methods:**

Recruitment and enrollment data were collected from a nationwide randomized controlled trial (RCT) of a Spanish-language smoking cessation intervention (N=1417). The effectiveness of each recruitment strategy was evaluated by computing the cost per participant (CPP), which is the ratio of direct cost over the number enrolled. More effective strategies yielded lower CPPs. Demographic and smoking-related characteristics of participants recruited via the two most effective strategies were also compared (n=1307).

**Results:**

Facebook was the most effective method (CPP=US $74.12), followed by TV advertisements (CPP=US $191.31), whereas public bus interior card advertising was the least effective method (CPP=US $642.50). Participants recruited via Facebook had lower average age (*P*=.008) and had spent fewer years in the United States (*P*<.001). Among the participants recruited via Facebook, a greater percentage of individuals had at least a high school education (*P*<.001) and an annual income above US $10,000 (*P*<.001). In addition, a greater percentage of individuals were employed (*P*<.001) and foreign born (*P*=.003). In terms of subethnicity, among the subjects recruited via Facebook, a lower percentage of individuals were of Mexican origin (*P*<.001) and a greater percentage of individuals were of Central American (*P*=.02), South American (*P*=.01), and Cuban (*P*<.001) origin.

**Conclusions:**

Facebook was the most effective method for recruiting Hispanic or Latinx smokers in the United States for this RCT. However, using multiple methods was necessary to recruit a more diverse sample of Spanish-preferring Hispanic or Latinx smokers.

## Introduction

Tobacco smoking is the leading cause of preventable death in the United States [1] and is associated with 4 of the 5 leading causes of death among Hispanics or Latinxs (cancer, heart disease, stroke, and diabetes) [2]. According to the US Census Bureau, Hispanics or Latinxs represent 18% of the US population and are considered the largest and the second fastest growing racial ethnic minority group, making tobacco cessation among this population a public health priority [3,4]. US Hispanic or Latinx adults generally report lower smoking rates (10%) than non-Hispanic Whites (15%) [5]. In addition, Hispanics or Latinxs smoke fewer cigarettes per day (CPD) and are more often nondaily smokers [6]. However, smoking prevalence has been found to vary by ethnic subgroup, with the highest prevalence among those of Puerto Rican (19% men and 16% women) and Mexican (15% men and 7% women) origins and the lowest prevalence among those of Dominican origin (6% total) [7].

Despite the significant burden of smoking-related morbidity and mortality, Hispanics or Latinxs are often underrepresented in clinical trials, including smoking cessation trials [6,8,9]. More than 10 years after the publication by the US Department of Health and Human Services [10] called for the development and evaluation of smoking cessation interventions for racial/ethnic minority groups, only a handful of trials have been conducted with Hispanics or Latinxs [11-16]. There is a scarcity of Spanish-language and evidence-based smoking cessation interventions tailored to this population [17,18]. Yet, 16 million Hispanics or Latinxs report speaking English less than “very well” or “not at all,” and another 11 million are bilingual but prefer to use Spanish [19,20].

Recruiting Hispanics or Latinxs for randomized trials can be challenging, especially among those who speak little or no English [21,22]. Additional factors that influence recruitment might also relate to acculturation responses, limited or no previous experience of participating in research, mistrust in study sponsors (eg, government sponsors), language and health literacy, and apprehension about deportation for undocumented immigrants [23]. Although several studies have examined recruitment strategies for enrolling smokers into randomized trials [24], there is a lack of research examining strategies for recruiting underrepresented smokers such as Hispanics or Latinxs [25-27]. Furthermore, few studies have examined the costs of different recruitment strategies [24]. Successfully recruiting Hispanic or Latinx smokers will allow researchers and public health organizations to develop and test more effective interventions and programs for this underserved population. This goal aligns with the national imperatives of inclusivity.

This paper describes the recruitment strategies, including number enrolled, direct cost per strategy, and cost per participant (CPP), for an ongoing randomized controlled trial (RCT) of Hispanic or Latinx smokers who prefer receiving their health information in Spanish [28]. Demographic and smoking-related characteristics of participants recruited via the two most effective strategies were also compared. These findings can be useful to maximize recruitment success for future studies and to inform resource allocation for reaching a large, diverse, and hard-to-reach group of smokers.

## Methods

### Overview of Randomized Trial

There is a lack of evidence-based, Spanish-language smoking cessation interventions available for Hispanics or Latinxs [17]. To address this gap, we developed a series of culturally relevant self-help booklets and pamphlets for Hispanic or Latinx smokers. This new, targeted intervention was developed by transcreating (translating + adapting) an existing English-language, validated self-help smoking cessation and relapse prevention intervention titled, *Forever Free*®: *Stop Smoking for Good* (SSFG) [29-31]. Using a multiphase qualitative approach, we successfully transcreated the *SSFG* intervention into a version tailored specifically for individuals who prefer health education materials in Spanish [31]. The Spanish-language version of *SSFG* is titled *Libre del cigarrillo, por mi familia y por mí: Guía para dejar de fumar* (*Free from Cigarettes, for my family and for me: Guide to quit smoking*). Details regarding booklet development and content are presented elsewhere [31].

In the ongoing RCT (trial registration: NCT02945787), the efficacy of the *Libre del Cigarrillo* intervention is compared with usual care, a Spanish-language smoking cessation booklet from the National Cancer Institute [28,32]. The 1417 Hispanic or Latinx smokers enrolled in the study were oversampled from Florida, 555 from Florida and 852 from the rest of the contiguous United States or Puerto Rico. Participants were randomly assigned to receive the *Libre del Cigarrillo* intervention or usual care and were asked to complete follow-up assessments every 6 months for 2 years. Participants received a US $20 gift card for each completed assessment. Details regarding procedures, baseline sample characteristics, and study design for the RCT are presented elsewhere [28].

#### Procedures

Recruitment for the RCT began in October 2016 and ended in June 2018. Study team members were bilingual, and all recruitment materials (eg, screening forms, advertisements) were in Spanish. Study advertisements included a toll-free number and a link to the study web page for potential participants to enter contact information. This study was reviewed and approved by the Chesapeake Institutional Review Board.

#### Measures

##### Eligibility Screening

Eligibility screenings were completed by phone and in Spanish by study staff located in Tampa, Florida. Inclusion criteria were age ≥18 years, smoking ≥5 tobacco cigarettes per week over the last year, not currently enrolled in a face-to-face smoking cessation program, and preference for receiving health education materials in Spanish. Smokers were excluded from participating in the study if they were unable to provide a valid US mailing address or if a member of their household was already enrolled in the study. During the screening, participants were asked how they heard about the study.

##### Baseline Assessment

The baseline survey was in Spanish and assessed sociodemographic characteristics (eg, race, Hispanic or Latinx subethnicity, education, etc), smoking history, nicotine dependence (Spanish version of the Fagerström Test for Nicotine Dependence) [33], and readiness to quit smoking (Contemplation Ladder) [34]. Other measures assessed the motivation to quit smoking, acculturation, familism, and affect.

##### Recruitment Strategies and Cost

We used the following recruitment strategies: Facebook, television (TV), website banners, bus signage, flyers, press releases, Craigslist posts, TV and radio interviews, and electronic mailing lists (Table 1). Table 2 presents a description of each recruitment method, the direct cost of implementation, the number of participants enrolled, and the CPP (the direct cost for implementing the strategy divided by the number of enrolled participants). The monetary value of staff time was not included in the cost calculations because the time spent on initiation and implementation was shared equally across the recruitment strategies. Similarly, any cost incurred by the Moffitt Cancer Center (MCC), such as materials (ie, printing supplies) and services (eg, shipping, public relations, and graphic design) were excluded from cost calculations. We only included expenses directly charged to the trial.

**Table 1:** Recruitment methods.

Methods	Descriptions	Geographic reach
Facebook advertisement	Paid advertisement of free smoking cessation materials containing smoking-related imagery, the study phone number, and the direct link to the web page. Advertisements targeted Hispanic or Latinx adults who showed interest in tobacco-related topics	Nationwide
TV advertisement	Paid 30-second video advertising of free smoking cessation materials as a part of a research study in Spanish. Advertisements ran on weekdays via Entravision Spanish-language TV stations	Tampa, Florida Orlando, Florida McAllen, Texas El Paso, Texas San Diego, California Albuquerque, New Mexico Santa Fe, New Mexico Hartford, Connecticut
Word of mouth	Participants told other smokers in their social network about the study without encouragement or compensation	N/A^a^
Website banner advertisement	Paid advertisement of free smoking cessation materials. The banners contained pictures of people smoking, the study phone number, and the direct link to the web page. Web-based advertisements were displayed on desktops and mobile devices and targeted Hispanic or Latinx adult smokers on multiple websites	Miami, Florida Puerto Rico
Public bus interior cards	Paid advertisement of free smoking cessation materials, the study phone number, and web page address. Advertisements were displayed on the interior of buses	Orlando, Florida
Flyers	CAB^b^ members distributed flyers at community clinics, health fairs, and recruitment events. Flyers publicized free smoking cessation materials and included the study phone number and web page address	Florida Texas New Mexico Puerto Rico California Arizona Colorado North Carolina Ohio Massachusetts
Radio and TV interviews	A team member was interviewed about the study. The show, called *Tu Salud Informa*, was hosted by a CAB member in Spanish	Puerto Rico
TV interview (Univision)	A team member was interviewed about the study on the show, *Pregúntale al Médico*	Tampa Bay area, Florida
Press releases	Stories featuring the RCT^c^ were distributed by the cancer center to local newspapers for the Hispanic Heritage Month and Lung Cancer Awareness Month	Tampa Bay area, Florida
Electronic mailing lists	An electronic newsletter including the study flyer was sent to bilingual community members in the Tampa Bay area and health care providers and community organizations in Puerto Rico	Tampa Bay area, Florida Puerto Rico
Craigslist	Free advertisement placed under the *community* section for 5 Florida counties	Hillsborough Lee Polk Miami-Dade Orange

^a^N/A: not applicable.

^b^CAB: community advisory board.

^c^RCT: randomized controlled trial.

**Table 2:** Recruitment cost of strategies directly charged to the project.

Recruitment methods	Direct cost to project, US $	Number of enrolled participants, n	Cost per enrolled participants^a^, US $	Duration, months	Number of enrolled participants per month^b^, n
Facebook advertisement	76,339	1030	74	16	64
Telivision advertisement	52,994	277	191	6	46
Website banner advertisement	4260	11	387	2	6
Public bus interior cards	1285	2	643	1	2

^a^Cost per enrolled participant=direct cost divided by the enrolled participant.

^b^Enrolled per month=enrolled participant divided by the duration.

#### TV Advertisement

The first strategy was a TV advertisement campaign. Entravision, an affiliate of the Univision and UniMás TV networks, created two 30-second videos (with a male and female voice-over) advertising no-cost smoking cessation materials, toll-free number, and web page. The first campaign ran from November 2016 to February 2017 in Texas, Florida, California, and New Mexico. The second campaign ran from June 2017 to July 2017 in Texas, Connecticut, and Massachusetts. Markets were selected based on the prevalence of smoking among adult Hispanics or Latinxs in each state. The videos were broadcast 1441 times by at least 13 TV stations. The advertisements initially ran Monday through Friday (weekends were substantially more expensive) but eventually focused on Wednesday through Friday because of greater response volumes. A limitation of TV advertising with Entravision was the company’s lack of affiliates in Miami and Puerto Rico, the 2 markets with a substantial Hispanic or Latinx population. The total cost for creating and placing TV advertisements was US $52,994. We screened 525 potential participants, 52.8% (277/525) of whom were enrolled.

#### Website Banner Advertising

To reach the Miami and Puerto Rico markets, we worked with Entravision’s Pulpo digital advertising unit, the leading Hispanic/Latinx advertising platform. A total of 5 versions of desktop and mobile banners were created that included a direct link to the study web page and content similar to the TV advertisements. The campaigns began in November 2016, but they were discontinued after 1 month because of a very low response rate (11 inquiries). A second digital campaign was run in Miami for 1 month (June 2017). Website banner advertisements were charged by impressions or the number of times the advertisements were shown. The costs were US $1500 (216,667 viewer impressions) for the first Miami campaign, US $2000 (300,000 viewer impressions) for the Puerto Rico campaign, and US $760 (110,000 viewer impressions) for the second Miami campaign. The total direct cost for the digital campaign was US $4620 or about US $0.07 per impression. A total of 16 potential participants were screened, and 11 (68%) participants were enrolled.

#### Facebook

We conducted a nationwide paid Facebook advertisement campaign to reach a broader segment of the target population. An advertising agency managed the campaign. Initially, users who self-identified as primarily Spanish-speaking and living in the United States were targeted. The campaign was also modified to target Hispanic bilingual users to extend the reach. The advertisements targeted users who *liked* or showed interest in tobacco-related topics using keywords (eg, cigarettes, quitting smoking, smoking, and tobacco smoking).

Facebook offered 1 format for the advertisements that included the use of 1 image per advertisement, a limited word count, the study phone number, and a link to the study web page. The research team used a different image, but identical text, for the 2 advertisements. The advertisements were reviewed and approved by Facebook [35]. After 11 months of launch, Facebook paused the campaign because the text did not meet its advertisement policies of attribution (targeting characteristics) [35]. Facebook restarted the campaign after revising and resubmitting text modifications, removing any indication that the advertisement was targeting known smokers.

Concurrent with the nationwide Facebook campaign, which displayed our advertisements to users in any US state and Puerto Rico, we targeted metropolitan areas within the following regions: Southwest (California, Texas, Arizona, New Mexico, Nevada, and Colorado); Northeast (Massachusetts, New York, New Jersey, and Connecticut); Midwest (Illinois); and Southeast United States (District of Columbia, Virginia, Georgia, and Florida). The Facebook advertisements ran from February 2017 to June 2018. The average daily spending was US $35.80, and the average cost per click was US $0.46. The cost of the advertising agency for project management over the course of the campaign was US $6751. Costs for the entire recruitment period totaled US $76,338. Of the 1686 screened individuals, 1030 (61.1%) were enrolled.

#### Public Transit Advertising

Direct Media USA (now Vector Media) developed and launched a public transit campaign in the Orlando, Florida, metropolitan area, where 31% of the population is Hispanic or Latinx [36]. MCC’s graphic design team created an 11”×28” color advertisement that was installed in the interior of 50 buses, reaching more than 100,000 passengers daily. The campaign ran from July to August 2017 and was discontinued because of a low response rate. The total cost was US $1285: a one-time setup fee of US $10.50 per display for 50 displays, a monthly media rate of US $12 per display, plus a shipping and processing charge of US $160. A total of 5 potential participants were screened, and 2 participants were enrolled.

#### Cultural Advisory Board and Community Partnerships

Recruitment was facilitated by a cultural advisory board (CAB) comprising researchers with experience in recruiting Hispanic or Latinx participants located throughout the Southwest United States and Puerto Rico. Full page color flyers publicizing no-cost smoking cessation materials were distributed at community clinics, health fairs, and recruitment events. The flyers included the study toll-free number and web page address. In addition to the CAB, 2 existing partnerships of academic and community-based organizations facilitated and supported the recruitment process: the Tampa Bay Community Cancer Network (TBCCN) and the Ponce School of Medicine-MCC Partnership in Puerto Rico. In the Tampa Bay area, flyers were distributed via a subset of the community-based organizations that comprise TBCCN. In Puerto Rico, in addition to flyers, information about the study was disseminated via TV (Telemundo/ABC affiliate) and radio (Radio Salud 1520 AM) interviews with a team member. One potential participant was screened from a TV interview. No participants were enrolled using flyers, TV, or radio interviews, and there was no direct cost to the study.

#### Television Interview

An interview was conducted in Univision Tampa Bay’s *Pregúntale al Médico (Ask the Physician)* with a team member about the study. The interview aired on WVEA-TV, an Entravision Communications local station serving the cities of Tampa and Saint Petersburg, FL. There was no direct cost for this service, which resulted in the screening of 2 potential participants and enrollment of 1 participant.

#### Additional Strategies

The study included an overrepresentation of participants from Florida to facilitate, in part, a biochemical verification of smoking status. Advertisements were placed on Craigslist for a month under the *community* section for 5 Florida counties (Hillsborough, Lee, Miami-Dade, Orange, and Polk). Electronic mailing lists were used to reach bilingual community members from the MCC’s catchment area and health care providers in the Tampa Bay area and Puerto Rico, respectively. In addition, press releases were distributed by MCC to newspapers in the Tampa Bay area for Hispanic Heritage Month and Lung Cancer Awareness Month in 2016. These strategies did not result in any enrolled participants, and there was no direct cost to the study.

#### Word of Mouth

A total of 62 enrolled participants indicated that they learned about the study through other people. Participants were not encouraged to tell other smokers about the study. Word of mouth was not a planned strategy, and there was no direct cost to the study.

### Analysis

To further inform recruitment strategies in future smoking cessation trials, analyses aimed to identify differences in demographics and smoking-related variables between participants recruited via Facebook versus TV advertisements, given that these were the recruitment methods that yielded the greatest number of participants. Comparisons were performed using two-tailed *t* tests for continuous variables and chi-square tests for categorical variables, with an alpha of .05. All statistical analyses were conducted using SPSS Statistics Version 25.0 (IBM Corporation).

## Results

### Participant Sample

For the RCT, 2387 smokers were screened. Of those, 2056 met the inclusion criteria, consented to participate, and were sent a baseline assessment. Of the 1467 who completed the baseline, 1417 were eligible and were randomized and enrolled in the study. Most participants (1307) were recruited via Facebook and TV advertisements. The 1030 participants recruited via Facebook represented 40 states from all US regions: South (n=648), West (n=191), Northeast (n=94), Midwest (n=44), and Puerto Rico (n=53).

### Enrollment and CPP

Facebook yielded the greatest number of enrolled participants with 72.69% (1030/1417) of the total sample, followed by TV with 19.54% (277/1417), and word of mouth with 4.38% (64/1417). Website banner advertisements and public bus interior cards yielded less than 1% (12/1417) of the enrolled participants, combined. Facebook had the lowest cost (CPP=US $74.12), followed by TV advertisements (CPP=US $191.31). Website banner advertisements (CPP=US $387.27) and public bus interior cards (CPP=US $642.50) were the most expensive recruitment methods. The computed CPPs for the strategies that were directly charged to the project are displayed in Table 2, along with the average number of enrolled participants per month.

### Facebook Versus TV

#### Demographic Variables

The characteristics of participants by recruitment strategy are presented in Table 3. Participants recruited via TV advertisements had a higher average age (t_1305_=−2.7; *P*=.008), and a higher percentage of them had less than a high school education (n=1270; χ^2^_1_*=*76.3; *P*<.001). In addition, a higher percentage of participants recruited via TV had an annual household income below US $10,000 (n=1227; χ^2^_1_*=*12.6; *P*<.001), and a lower percentage of them were employed (n=1274; χ^2^_1_*^=^*35.9; *P*<.001). Participants recruited via TV advertisements had a higher average number of years living in the United States (t_1207_=−6.6; *P*<.001) and a lower percentage of foreign births (n=1299; χ^2^_1_=8.8; *P*=.003). In terms of subethnicity, a higher percentage of participants recruited via TV advertisements were of Mexican/Mexican American origin (n=1300; χ^2^_1_=54.4; *P*<.001) and a lower percentage of them were of Central American origin (n=1300; χ^2^_1_=5.6; *P*=.02), South American origin (n=1300; χ^2^_1_=6.6; *P*=.01), and Cuban origin (n=1300; χ^2^_1_=41.4; *P*<.001). Finally, participants recruited via TV advertisements had a higher percentage of those not reporting race (n=1307; χ^2^_1_=16.7; *P*<.001).

#### Other Variables

There were no statistically significant differences in the recruitment method for acculturation or any of the smoking-related variables.

**Table 3:** Participant characteristics by the recruitment method (n=1307).

Characteristics		Facebook (n=1030)	Television (n=277)	*P* value
Age (years), mean (SD)		49.4 (11.6)	51.5 (12.0)	.008
**Age group (years), n (%)**				.12
	18-29	51 (5.0)	12 (4.3)	
	30-39	171 (16.6)	34 (12.3)	
	40-49	249 (24.2)	67 (24.2)	
	50-59	361 (35.0)	93 (33.6)	
	≥60	198 (19.2)	71 (25.6)	
Sex (women), n (%)		490 (47.6)	143 (51.6)	.23
Education (<high school diploma), n (%)		251 (25.0)	141 (52.8)	<.001
Employed, n (%)		631 (62.7)	113 (42.3)	<.001
Annual income per household <US $10,000, n (%)		371 (38.1)	128 (50.4)	<.001
Marital status (married or cohabiting), n (%)		479 (46.9)	139 (50.2)	.33
**Race, n (%)**				.001
	White	486 (47.2)	109 (39.4)	.10
	Black or African American	34 (3.3)	2 (0.7)	.06
	American Indian or Alaska Native	32 (3.1)	6 (2.2)	.82
	Asian	1 (0.1)	1 (0.4)	.31
	Native Hawaiian or Other Pacific Islander	24 (2.3)	4 (1.4)	.80
	Multiple races	40 (3.9)	6 (2.2)	.44
	Not reported	413 (40.1)	149 (53.8)	<.001
**Subethnicity, n (%)**				<.001
	Puerto Rican	163 (15.9)	53 (19.3)	.17
	Central American	69 (6.7)	8 (2.9)	.02
	Mexican or Mexican American	315 (30.7)	150 (54.2)	<.001
	South American	99 (9.6)	13 (4.7)	.01
	Cuban	254 (24.8)	19 (6.9)	<.001
	Dominican	28 (2.7)	6 (2.2)	.62
	Other	14 (1.4)	3 (1.1)	.73
	More than one subethnicity	84 (8.2)	22 (8.0)	.93
Born outside the United States, n (%)		812 (78.8)	195 (70.4)	.003
Years in the United States (for foreign born)^a^, mean (SD)		14.1 (11.7)	20.4 (13.0)	<.001
Acculturation (SASH)^b^, mean (SD)		19.7 (6.3)	19.0 (6.5)	.08
**Smoking-related variables**				
	Smoke daily, n (%)	970 (94.2)	253 (91.3)	.12
	E-cigarettes^c^, n (%)	45 (4.4)	11 (4.0)	.80
	Years of smoking, mean (SD)	27.8 (12.6)	29.4 (13.8)	.09
	CPD^d^, mean (SD)	14.9 (8.5)	14.0 (7.7)	.09
	FTND^e^, mean (SD)	4.9 (2.4)	4.9 (2.3)	.90
	Contemplation ladder, mean (SD)	6.9 (2.8)	7.0 (2.9)	.49

^a^Only includes those who were not born in the United States or Puerto Rico.

^b^SASH: Short Acculturation Scale for Hispanics.

^c^e-cigarettes: electronic cigarettes.

^d^CPD: cigarettes per day.

^e^FTND: Fagerström Test for Nicotine Dependence.

## Discussion

### Principal Findings

A greater representation of Hispanic or Latinx individuals is needed in smoking cessation trials. Moreover, given the heterogeneity of the Hispanic or Latinx population, it may be necessary to analyze which recruitment strategies successfully contributed to the accrual of a diverse sample. This study aimed to evaluate the methods used to recruit Spanish-speaking Hispanic or Latinx smokers in terms of CPP and enrollment success. The results will inform the development and implementation of effective recruitment strategies for future studies.

The ongoing RCT used a combination of mass media (ie, TV advertisements, press releases, and TV and radio interviews); social media (ie, Facebook); and internet-based (ie, banner advertisements) and community outreach strategies. A total of 3 recruitment methods accounted for the majority of enrolled participants: Facebook, TV, and word of mouth. Facebook was ultimately the most effective recruitment method based on CPP (US $74 per participant), and it also yielded the highest average enrollees per month (n=64). TV advertisements, the original recruitment method, yielded the second highest number of participants at about 46 enrollees per month and were the second most effective strategy at US $190 CPP. There are several reasons we believe Facebook was most effective in reaching our intended population. Unlike TV advertisements, which can be very expensive in markets with a high density of Hispanics or Latinxs (eg, Miami, New York City, and Puerto Rico), Facebook advertising campaigns can have a national reach with an affordable cost regardless of the city, state, or Hispanic or Latinx population share. We were also able to further target our advertising using interest-based keywords (eg, cigarettes, quitting smoking, smoking, and tobacco smoking), which is a unique benefit of using this platform as compared with TV. Another reason Facebook was the most effective method for the current RCT is that this platform is a trusted media source among Hispanics or Latinxs. Evidence suggests that Hispanics or Latinxs are receptive to advertising via Facebook, and compared with the other cultural groups, Hispanics or Latinxs have been found to be more inclined to share content with their extended networks [37].

In comparison with mass media, social media/web-based advertisements can be highly targeted, thus increasing the chances of reaching the intended population, which should translate to more eligible participants. However, Facebook policies targeting personal attributes changed midstudy. The original advertisement stated, “Are *you* interested in quitting smoking?” with the assumption that the social media user was a smoker. Following the policy change, less emphasis was placed on the user’s personal attributes and more emphasis on the availability of materials to quit smoking at no cost. An advertisement introduced later stated, “Quit smoking help available at no cost!” Furthermore, the process of revision and approval for Facebook advertisements was lengthy and iterative, which contributed to unanticipated delays in recruitment. Future researchers may consider consulting with Facebook earlier in advertisement development to reduce potential delays.

Offline campaigns, such as TV advertisements, usually resulted in call volumes that peaked immediately following the release of the advertisement. Facebook advertisements typically resulted in a steady response rate. Another advantage of Facebook advertisements was that they linked interested individuals directly to the study web page.

It is important to consider that Hispanics or Latinxs are among the leading social media users and the fastest growing ethnic group on Facebook in the United States. In 2015, 75% of Hispanic or Latinx adult internet users were on Facebook (compared with 45% in 2010) [38,39]. Furthermore, a recent report on TV viewership found that engagement with Spanish-language TV and the household reach of Spanish networks in the United States have decreased in recent years [40]. However, mass media (ie, TV and radio) have shown success in reaching the Hispanic or Latinx audience [39,41]. Accordingly, TV advertisements still played an essential role in ensuring that we recruited a diverse sample. Participants recruited via TV advertisements were 2 years older on average and had lower socioeconomic attainment (ie, education, income, and employment) than those recruited via Facebook. As such, utilizing methods besides Facebook is an essential strategy to ensure that a wider diversity of socioeconomic status is included. Ensuring that this subgroup is captured in smoking cessation trials is of particular importance because research demonstrates a higher prevalence of smoking among lower socioeconomic status groups, and they greatly bear the burden of tobacco-related health disparities [42].

Furthermore, using Facebook in addition to TV was essential in enrolling a diverse sample of Hispanic or Latinx subgroups. A higher percentage of Mexican/Mexican American participants were recruited via TV advertisements, whereas a higher percentage of Central American, South American, and Cuban participants were recruited from Facebook. The greater representation of Mexican Americans is possibly a reflection of heavier TV advertising in the Southwest. Given the variations within the Hispanic or Latinx population in terms of lifestyle, socioeconomic characteristics, access to health care, and smoking patterns, it is important to develop recruitment strategies to ensure representation from all countries of origin [43-47]. A more representative sample allows for comparisons across groups and increases the potential generalizability of these findings. It is evident that Facebook and TV advertisements attracted participants with different countries of origin into the RCT.

Unexpectedly, word of mouth was the method that yielded the third highest number of participants, even though we did not encourage or compensate for referrals. Brodar et al [26] also found that word of mouth was a free method that was highly effective in yielding enrolled smokers. Receiving free smoking cessation materials and compensation for completing assessments may have motivated participants to tell their family members and friends about the study. However, researchers should be cautious about participants within the same social network being randomized to different intervention arms because of the potential for cross-contamination. Another unexpected finding was the poor performance of the flyers. However, this is consistent with past research showing that Hispanic or Latinx smokers were less likely to be recruited by flyers [26,48].

Among paid recruitment methods, website banners and bus signs yielded very few participants at a very high cost, consistent with previous reports [26]. Website banner advertisements may be more effective than physical advertisements because they provide potential participants with easier and more immediate access to the study web page.

### Limitations

This study has some limitations that should be noted. First, there is a possibility of misclassification of recruitment methods by participants. However, only a negligible number of screened individuals (n*=*48) and enrolled participants (n=34) endorsed methods that were not used in their geographic region. Second, given the broad reach of the mass media campaign, and the fact that we used a variety of methods, it is possible that participants were exposed to more than one advertising modality. Third, only a single social media platform (ie, Facebook) was used. Twitter and Instagram, with about 10 and 13 million Hispanic or Latinx adult users, respectively, are other social media sites that hold great potential for recruiting participants from this population [38]. However, as stated previously, Facebook is the most popular social media platform among Hispanic or Latinx adults with 28 million users, more than Instagram and Twitter combined [38]. Finally, the representativeness of this sample to the population of US Spanish-language preferred, treatment-seeking Hispanic or Latinx smokers is difficult to know. On average, this sample comprised older, heavier smoking, and more daily smokers than national samples of Hispanic or Latinx smokers [43]. However, these demographics are not unusual for a treatment-seeking subpopulation of smokers.

### Conclusions

Overall, successfully recruiting a socioeconomically and ethnically diverse sample of Hispanic or Latinx smokers is necessary to increase the access to cessation trials and the generalizability of study findings, in response to national calls for more inclusivity in clinical research [49]. Using multiple strategies, we recruited a large national sample of Hispanic or Latinx smokers representing diverse ethnic subgroups from this population. The utilization of social media was an effective method to recruit participants into an RCT and improve the inclusion of a historically underrepresented population. To the best of our knowledge, no other smoking cessation trial with Hispanic or Latinx smokers has reported on using Facebook as a recruitment strategy [50]. Social media use is rapidly growing among Hispanic or Latinx adults and holds great potential for the recruitment of study participants. Facebook is a low-cost, effective method to recruit Hispanic or Latinx smokers. However, mass media campaigns, specifically via TV, are still needed to reach a segment of the Hispanic or Latinx population that is at a greater socioeconomic disadvantage. Researchers aiming to enroll a representative sample of Hispanic or Latinx smokers should consider both recruitment methods.

## Abbreviations

CAB: cultural advisory board
CPD: cigarettes per day
CPP: cost per participant
MCC: Moffitt Cancer Center
RCT: randomized controlled trial
SSFG: Stop Smoking for Good
TBCCN: Tampa Bay Community Cancer Network

## References

[1] (2014). 2014 Surgeon General's Report: The Health Consequences of Smoking—50 Years of Progress. Centers for Disease Control and Prevention.

[2] (2017). Leading Causes of Death and Numbers of Deaths, by Sex, Race, and Hispanic Origin: United States, 1980 and 2016. Centers for Disease Control and Prevention.

[3] (2017). Facts for Features: Hispanic Heritage Month 2017. United States Census.

[4] Flores A (2017). How the US Hispanic Population is Changing. Pew Research Center.

[5] Wang TW, Asman K, Gentzke AS, Cullen KA, Holder-Hayes E, Reyes-Guzman C, Jamal A, Neff L, King BA (2018). Tobacco product use among adults - United States, 2017. MMWR Morb Mortal Wkly Rep.

[6] Trinidad D, Pérez-Stable EJ, Emery S, White M, Grana R, Messer K (2009). Intermittent and light daily smoking across racial/ethnic groups in the United States. Nicotine Tob Res.

[7] Miller KD, Goding Sauer A, Ortiz AP, Fedewa SA, Pinheiro PS, Tortolero-Luna G, Martinez-Tyson D, Jemal A, Siegel RL (2018). Cancer statistics for Hispanics/Latinos, 2018. CA Cancer J Clin.

[8] Ford ME, Siminoff LA, Pickelsimer E, Mainous AG, Smith DW, Diaz VA, Soderstrom LH, Jefferson MS, Tilley BC (2013). Unequal burden of disease, unequal participation in clinical trials: solutions from African American and Latino community members. Health Soc Work.

[9] George S, Duran N, Norris K (2014). A systematic review of barriers and facilitators to minority research participation among African Americans, Latinos, Asian Americans, and Pacific Islanders. Am J Public Health.

[10] Clinical Practice Guideline Treating Tobacco Use and Dependence 2008 Update Panel‚ Liaisons‚ and Staff (2008). A clinical practice guideline for treating tobacco use and dependence: 2008 update. A US public health service report. Am J Prev Med.

[11] Stanton CA, Papandonatos GD, Shuter J, Bicki A, Lloyd-Richardson EE, de Dios MA, Morrow KM, Makgoeng SB, Tashima KT, Niaura RS (2015). Outcomes of a tailored intervention for cigarette smoking cessation among Latinos living with HIV/AIDS. Nicotine Tob Res.

[12] de Dios MA, Cano M, Vaughan E, Childress SD, McNeel MM, Harvey LM, Niaura RS (2019). A pilot randomized trial examining the feasibility and acceptability of a culturally tailored and adherence-enhancing intervention for Latino smokers in the US. PLoS One.

[13] Asfar T, Caban-Martinez AJ, McClure LA, Ruano-Herreria EC, Sierra D, Gilford Clark G, Samano D, Dietz NA, Ward KD, Arheart KL, Lee DJ (2018). A cluster randomized pilot trial of a tailored worksite smoking cessation intervention targeting Hispanic/Latino construction workers: intervention development and research design. Contemp Clin Trials.

[14] Borrelli B, McQuaid EL, Novak SP, Hammond SK, Becker B (2010). Motivating Latino caregivers of children with asthma to quit smoking: a randomized trial. J Consult Clin Psychol.

[15] Cabriales JA, Cooper TV, Salgado-Garcia F, Naylor N, Gonzalez E (2012). A randomized trial of a brief smoking cessation intervention in a light and intermittent Hispanic sample. Exp Clin Psychopharmacol.

[16] Pollak KI, Lyna P, Bilheimer AK, Gordon KC, Peterson BL, Gao X, Swamy GK, Denman S, Gonzalez A, Rocha P, Fish LJ (2015). Efficacy of a couple-based randomized controlled trial to help Latino fathers quit smoking during pregnancy and postpartum: the Parejas trial. Cancer Epidemiol Biomarkers Prev.

[17] Webb MS, Rodríguez-Esquivel D, Baker EA (2010). Smoking cessation interventions among Hispanics in the United States: a systematic review and mini meta-analysis. Am J Health Promot.

[18] Báezconde-Garbanati L, Beebe LA, Pérez-Stable EJ (2007). Building capacity to address tobacco-related disparities among American Indian and Hispanic/Latino communities: conceptual and systemic considerations. Addiction.

[19] Krogstan J, Stepler R, Lopez M (2015). English Proficiency on the Rise Among Latinos. Pew Research Center.

[20] Krosgstad J, Gonzalez-Barrera A (2015). A Majority of English-speaking Hispanics in the US Are Bilingual. Pew Research Center.

[21] Aguirre TM, Koehler AE, Joshi A, Wilhelm SL (2018). Recruitment and retention challenges and successes. Ethn Health.

[22] Felsen CB, Shaw EK, Ferrante JM, Lacroix LJ, Crabtree BF (2010). Strategies for in-person recruitment: lessons learned from a New Jersey primary care research network (NJPCRN) study. J Am Board Fam Med.

[23] Sha M, McAvinchey G, Quiroz R, Moncada J (2017). Successful techniques to recruit Hispanic and Latino research participants. Surv Pract.

[24] Belisario JS, Bruggeling MN, Gunn LH, Brusamento S, Car J (2012). Interventions for recruiting smokers into cessation programmes. Cochrane Database Syst Rev.

[25] Thompson TP, Greaves CJ, Ayres R, Aveyard P, Warren FC, Byng R, Taylor RS, Campbell JL, Ussher M, Michie S, West R, Taylor AH (2015). Lessons learned from recruiting socioeconomically disadvantaged smokers into a pilot randomized controlled trial to explore the role of exercise assisted reduction then stop (EARS) smoking. Trials.

[26] Brodar KE, Hall MG, Butler EN, Parada H, Stein-Seroussi A, Hanley S, Brewer NT (2016). Recruiting diverse smokers: enrollment yields and cost. Int J Environ Res Public Health.

[27] Webb MS, Seigers D, Wood EA (2009). Recruiting African American smokers into intervention research: relationships between recruitment strategies and participant characteristics. Res Nurs Health.

[28] Medina-Ramírez P, Sutton SK, Martínez U, Meade CD, Byrne MM, Brandon KO, Meltzer LR, Gonzales FM, Brandon TH, Simmons VN (2019). A randomized controlled trial of a smoking cessation self-help intervention for Spanish-speaking Hispanic/Latinx smokers: study design and baseline characteristics. Contemp Clin Trials.

[29] Brandon TH, Simmons VN, Sutton SK, Unrod M, Harrell PT, Meade CD, Craig BM, Lee J, Meltzer LR (2016). Extended self-help for smoking cessation: a randomized controlled trial. Am J Prev Med.

[30] Simmons VN, Sutton SK, Meltzer LR, Unrod M, Meade CD, Brandon TH (2018). Long-term outcomes from a self-help smoking cessation randomized controlled trial. Psychol Addict Behav.

[31] Piñeiro B, Díaz DR, Monsalve LM, Martínez U, Meade CD, Meltzer LR, Brandon KO, Unrod M, Brandon TH, Simmons VN (2018). Systematic transcreation of self-help smoking cessation materials for Hispanic/Latino smokers: improving cultural relevance and acceptability. J Health Commun.

[32] (2011). Guía: Viva De Forma Más Saludable Para Usted Y Su Familia. SmokeFree.

[33] Becoña E, Vázquez FL (1998). The Fagerström test for nicotine dependence in a Spanish sample. Psychol Rep.

[34] Biener L, Abrams DB (1991). The contemplation ladder: validation of a measure of readiness to consider smoking cessation. Health Psychol.

[35] Advertising Policies. Facebook.

[36] Orlando-Kissimmee-Sanford, FL Metro Area. Census Reporter.

[37] (2014). Digital Diversity: A Closer Look at US Hispanics. Facebook for Business.

[38] Duggan M, Ellison N, Lampe C, Lenhart A, Madden M (2015). Social Media Update 2014. Pew Research Center.

[39] (2012). Cultural Insights; Communicating With Hispanics/latinos. CDC Stacks.

[40] Rice C (2018). Are Hispanic Viewers Watching Less Spanish Language TV?. Samba TV.

[41] Sonderup L (2010). Hispanic Marketing: A Critical Market Segment. Advertising & Marketing Review.

[42] (2017). The Epidemiology of Tobacco-Related Health Disparities. Division of Cancer Control and Population Sciences: DCCPS.

[43] Kaplan RC, Bangdiwala SI, Barnhart JM, Castañeda SF, Gellman MD, Lee DJ, Pérez-Stable EJ, Talavera GA, Youngblood ME, Giachello AL (2014). Smoking among US Hispanic/Latino adults: the Hispanic community health study/study of Latinos. Am J Prev Med.

[44] Blanco L, Garcia R, Pérez-Stable EJ, White MM, Messer K, Pierce JP, Trinidad DR (2014). National trends in smoking behaviors among Mexican, Puerto Rican, and Cuban men and women in the United States. Am J Public Health.

[45] Morales LS, Lara M, Kington RS, Valdez RO, Escarce JJ (2002). Socioeconomic, cultural, and behavioral factors affecting Hispanic health outcomes. J Health Care Poor Underserved.

[46] Mothel S, Patten E (2012). The 10 Largest Hispanic Origin Groups: Characteristics, Rankings, Top Counties. Pew Research Center.

[47] (2019). Profile: Hispanic/Latino Americans. The Office of Minority Health (OMH).

[48] García AA, Zuñiga JA, Lagon C (2017). A personal touch: the most important strategy for recruiting Latino research participants. J Transcult Nurs.

[49] (2017). NIH Policy and Guidelines on The Inclusion of Women and Minorities as Subjects in Clinical Research. NIH Central Resource for Grants and Funding Information.

[50] Hudnut-Beumler J, Po'e E, Barkin S (2016). The use of social media for health promotion in Hispanic populations: a scoping systematic review. JMIR Public Health Surveill.

